# 

**Published:** 1904-11

**Authors:** 


### BOOKS RECEIVED.

Saunders's Question Compends. Essentials of Nervous Diseases and Insanity: their Symptoms and Treatment. By John C. Shaw, M.D., late Clinical Professor of Diseases of the Mind and Nervous System, Long Island College Hospital Medical School. Fourth edition, thoroughly revised. By Smith Ely Jelliffe, Ph.G., M.D., Clinical Assistant, Columbia University. Duodecimo volume of 196 pages, illustrated. Philadelphia, New York, London: W. B. Saunders & Company. 1904. (Cloth, $1.00 net.)

Saunders's Question Compends. Essentials of Bacteriology. By M. V. Ball, M.D., formerly Resident Physician at the German Hospital, Philadelphia. Fifth edition, thoroughly revised. By Karl M. Vogel, M.D., Assistant Pathologist at the College of Physicians and Surgeons (Columbia University), New York City. Duodecimo volume of 343 pages, with 96 illustrations, some in colors, and six plates. Philadelphia, New York, London: W. B. Saunders & Company. 1904. (Cloth, $1.00 net.)

Saunders's Question Compends. Essentials of Anatomy; including the Anatomy of the Viscera. By Charles B. Nancrede, M.D., Professor of Surgery and Clinical Surgery in the University of Michigan, Ann Arbor. Seventh edition, thoroughly revised. Duodecimo volume of 419 pages, illustrated. Philadelphia, New York, London: W. B. Saunders & Company. 1904. (Cloth, $1.00 net.)

Saunders's Question Compends. Essentials of Materia Medica and Prescription Writing. By Henry Morris, M.D., College of Physicians, Philadelphia. Sixth edition, thoroughly revised. By W. A. Bastedo, Ph.G.,

M.D., Tutor of Materia Medica and Pharmacology at the Columbia University (College of Physicians and Surgeons), New York City. Duodecimo volume of 295 pages. Philadelphia, New York, London: W. B. Saunders & Company. 1904. (Cloth, $1.00 net.)

Blood Pressure. As Affecting Heart, Brain, Kidneys and General Circulation. By Louis F. Bishop, M.D., Physician to the Lincoln Hospital, New York. One hundred and twelve pages. New York: E. B. Treat & Company. 1904. (Price, $1.00.)

The Medical Directory of New York, New Jersey and Connecticut. Published by the New York State Medical Association. Volume VI. 1904-1905.

Davis's Obstetrics. A Treatise on Obstetrics. For Students and Practitioners. By Edward P. Davis, A.M., M.D., Professor of Obsetrics in Jefferson Medical College; Professor of Obsetrics and Pediatrics in the Philadelphia Polyclinic, etc. New (second) edition, thoroughly revised and much enlarged. Octavo, 800 pages, with 274 engravings and 39 full-page plates in colors and monochrome. New York and Philadelphia: Lea Brothers & Company. 1904. (Cloth, $5.00 net; leather, $6.00 net.)

Lectures to General Practitioners on the Diseases of the Stomach and Intestines, by Boardman Reed, M.D., Professor of Diseases of the Gastrointestinal Tract, Temple College, Philadelphia. Octavo, 1021 pages. Illustrated. New York: E. B. Treat & Company. 1904. (Price, $5.00.)

A Manual of Experimental Physiology. By Winfield S. Hall, Ph.D., M.D., Professor of Physiology, Northwestern University Medical School, Chicago. Octavo, 245 pages. Illustrated. New York and Philadelphia: Lea Brothers & Company. 1904. (Price, $2.75.)

Practical Dietetics. With reference to Diet in Disease. By Alida Frances Pattee, Instructor in Dietetics, Bellevue Training School for Nurses. Second edition. Duodecimo, 311 pages. Published by the author, 52 West 39th street, New York. (Price, $1.00.)

Beauty through Hygiene. By Emma E. Walker, M.D. Small duodecimo, 301 pages. New York: A. S. Barnes & Company. 1904. (Price, $1.00.)

Lea's Series of Medical Epitomes. Toxicology. By Edwin Welles Dwight, M.D., Instructor in Legal Medicine, Harvard University. Duodecimo, 298 pages. Series edited by V. C. Pedersen, M.D., Instructor in Surgery at the New York Polyclinic Medical School and Hospital. New York, Philadelphia: Lea Brothers & Company. 1904. (Price, $1.00.)

International Clinics. A Quarterly of Illustrated Clinical Lectures and especially prepared articles on Treatment, Medicine, Surgery, Neurology, Pediatrics, Obstetrics, Gynecology, Orthopedics, Pathology, Dermatology, Ophthalmology, Otology, Rhinology, Laryngology, Hygiene and other topics of interest to students and practitioners. By leading members of the medical profession throughout the world. Edited by A. O. J. Kelly, A.M., M.D., Philadelphia. Volume III. Fourteenth series. Philadelphia: J. B. Lippincott Company. 1904. (Cloth, $2.00.)

Handbook of the Anatomy and Diseases of the Eye and Ear. For Students and Practitioners. By D. B. St. John Roosa, M.D., LL.D., Professor of Diseases of the Eye and Ear in the New York Post-graduate Medical School, and A. Edward Davis, A.M., M.D., Professor of Diseases of the Eye in the New York Post-graduate Medical School. Duodecimo, 300 pages. Philadelphia: F. A. Davis Company. 1904. (Price, $1.00 net.)

Diseases of the Nose, Throat, and Ear and their Accessory Cavities. By Seth Scott Bishop, M.D., D.C.L., LL.D., Professor of Diseases of the

Nose, Throat, and Ear in the Illinois Medical College. Third edition, revised and enlarged. Illustrated with 94 colored lithographs and 230 additional illustrations. Octavo, 564 pages. Philadelphia: F. A. Davis Company. (Price: cloth, $4.00; sheep or half-russia, $5.00 net.)

Surgery of the Prostate, Pancreas, Diaphragm, Spleen, Thyroid, and Hydrocephalus. A Historical Review. By Benjamin Merrill Ricketts, M.D., Cincinnati. 1904.

Refraction and How to Refract. By James Thorington, M.D., Professor of Diseases of the Eye in the Philadelphia Polyclinic. Third edition. Duodecimo, 314 pages. Illustrated. Philadelphia: P. Blakiston's Son & Company. 1904. (Price, $1.50.)

Manual of Physiological and Clinical Chemistry. By Elias H. Bartley, B.S., M.D., Ph.G. Professor of Chemistry, Toxicology and Pediatrics in the Long Island College Hospital. Second edition, revised and enlarged. Duodecimo, 188 pages. Illustrated. Philadelphia: P. Blakiston's Son & Company. 1904. (Price, $1.00.)

A System of Practical Surgery. By Prof. E. von Bergman, M.D., Berlin, Prof. P. von Bruns, M.D., Tübingen, and Prof. J. von Mikulicz, M.D., Breslau. Volume V. Surgery of the Pelvis and Genitourinary Organs. Translated and edited by William T. Bull, M.D., Professor of Surgery, College of Physicians and Surgeons, New York, and Edward Milton Foote, M.D., Instructor in Surgery. Royal octavo, 789 pages. Illustrated. New York and Philadelphia: Lea Brothers and Company. 1904. (Price: cloth, $6.00; leather, $7.00; half-morocco, $8.50 net.)

The Surgical Treatment of Bright's Disease. By George M. Edebohls, A.M., M.D., LL.D., Professor of the Diseases of Women in the New York Post-graduate Medical School and Hospital. Octavo, 327 pages. New York: Frank F. Lisiecki, Publisher. 1904.

A Textbook of Histology. By Frederick R. Bailey, A.M., M.D., Adjunct Professor of Normal Histology, College of Physicians and Surgeons, New York. Octavo, 481 pages. Illustrated. New York: William Wood & Company. 1904. (Price, $3.00.)

The Practical Medicine Series of Year Books. Ten volumes. Issued under the general editorial charge of Gustavus P. Head, M.D., Professor of Laryngology and Rhinology in the Chicago Post-Graduate Medical School. Volume VIII. Materia Medica, Therapeutics, etc. Edited by George F. Butler and Collaborators. Chicago: The Year Book Publishers. 1904. (Price, $1.00; entire series, $5.50, payable in advance.)

The Practical Medicine Series of Year Books. Ten volumes. Issued under the general editorial charge of Gustavus P. Head, M.D., Professor of Laryngology and Rhinology in the Chicago Post-Graduate Medical School. Volume IX. Physiology, Pathology, etc. Edited by W. A. Evans and collaborators. Chicago: The Year Book Publishers. 1904. (Price, $1.00; entire series, $5.50, payable in advance.)

The Practical Medicine Series of Year Books. Ten volumes. Issued under the general editorial charge of Gustavus P. Head, M.D., Professor of Laryngology and Rhinology in the Chicago Post-Graduate Medical School. Volume X. Skin and Venereal Diseases; Nervous and Mental Diseases. Edited by W. L. Baum and Hugh T. Patrick. Chicago: The Year Book Publishers. 1904. (Price, $1.00; entire series, $5.50, payable in advance.)

The Urine and Clinical Chemistry of the Gastric Contents, the Common Poisons, and Milk. By J. W. Holland, M.D., Professor of Medical Chemistry and Toxicology, Jefferson Medical College, Philadelphia. Seventh edition, revised and enlarged, with 41 illustrations. Philadelphia: P. Blakiston's Son & Co. 1904.

